# An Updated Understanding of the Current Emerging Respiratory Infection: COVID-19

**DOI:** 10.1155/2020/6870512

**Published:** 2020-10-27

**Authors:** Hai Yuan, Xiaoguang Cao, Xiaoqi Ji, Fangbing Du, Jiawei He, Xuan Zhou, Yanghu Xie, Yu Zhu

**Affiliations:** ^1^Department of Rehabilitation Medicine, The Second People's Hospital of Hefei City, Hefei 230011, China; ^2^Department of Intensive Care Unit, The Second People's Hospital of Hefei City, Hefei 230011, China; ^3^Department of Respiratory Medicine, The Second People's Hospital of Hefei City, Hefei 230011, China; ^4^Department of Radiology, The Second People's Hospital of Hefei City, Hefei 230011, China; ^5^Department of Laboratory Medicine, The Second People's Hospital of Hefei City, Hefei 230011, China; ^6^School of Public Health, Wannan Medical College, Wuhu 241002, China

## Abstract

According to the World Health Organization (WHO), the COVID-19 pandemic has been declared as a priority disease. Some patients with COVID-19 had symptoms of multiple organ failure and death. The published articles on COVID-19 infection were reviewed. The origin of SARS-CoV-2 is still not completely established. Person-to-person transmission via droplets, probable aerosols, or close contact is considered as the main mode of transmission. With increased mortality due to SARS-CoV-2, valuable clinical indicators or treatments should be further identified and summarized. CT scanning plays an important role in the diagnosis and evaluation of COVID-19 in asymptomatic patients or those with initially negative RT-PCR results. No specific antiviral therapy is recommended, except the main supportive treatments, and effective measures should be taken into consideration to protect important organs and prevent the development of acute respiratory distress syndrome (ARDS) in patients with severe infection.

## 1. Introduction

A cluster of patients with pneumonia of unknown etiology was detected in Wuhan, China, in December 2019 [1]. Both the Chinese Center for Disease Control and Prevention [2–4] and the World Health Organization (WHO) [5–7] confirmed the identity of a novel coronavirus (2019-nCoV) on 07 January 2020. This virus has been isolated and verified from the infected patients [8] and named Severe Acute Respiratory Syndrome-coronavirus-2 (SARS-CoV-2) [9] by the International Committee on Taxonomy of Viruses. The disease was named as coronavirus disease (COVID-19) by the WHO [10, 11]. On 14 March 2020, 81021 confirmed cases and 3194 deaths have been reported in China. Patients with COVID-19 infection have also been detected and reported in Thailand (Jan 13), Japan (Jan 15), South Korea (Jan 20), the United States (Jan 23), Vietnam (Jan 24), Republic of Singapore (Jan 24), France (Jan 25), Nepal (Jan 25), Australia (Jan 25), Malaysia (Jan 26), and so on [12]. On 30 Jan 2020, following the advice of the Emergency Committee, the WHO Director-General announced the outbreak of COVID-19 as a public health emergency of international concern [13, 14]. The epidemic transmission in China has been effectively controlled currently. COVID-19 has gradually spread outside China, and its situation still remained to be intolerable in Japan, South Korea, Italy, Iran, and so on [12]. There is a possible epidemiological association of this disease with seafood and wild animal markets, thought to be the location of the initial outbreak in Wuhan. Most of the people who lived or worked at the market showed association with exposure to the virus reservoir, indicating a possible condition of zoonoses. However, the origins and hosts of SARS-CoV-2 are still not clarified. The patients with COVID-19 have been discharged from hospitals, and its pathogenesis is gradually identified; however, the diagnosis and treatments of viral pneumonia should be further summarized. In this review, the pandemic situation of COVID-19 is presented, discussing the current state to combat emerging coronavirus infections. The clinical features, diagnosis, and treatments associated with viral pneumonia are discussed to assist physicians in timely and accurate diagnoses and treat infected patients.

## 2. Epidemiology

### 2.1. Origin

Initial cases have shown direct or indirect contact history with the original seafood market, and it is considered as the original place of the outbreak of COVID-19. It is unknown erca

this novel coronavirus is originated from the sold wild animals of Huanan seafood market.

SARS-CoV-2 is an enveloped single-stranded plus stranded RNA virus with a diameter of 60-140 nm [5]. The virus was isolated from a patient on 7 January 2020, and the genomic sequence of SARS-CoV-2 was released by Chinese researchers on 10 January 2020 with the assistance of Global Initiative on Sharing All Influenza Data (GISAID) [15]. The SARS-CoV-2 is a new beta coronavirus that belongs to group 2B based on genome sequence analysis, but its S protein sequence differed from that of SARS-CoV [16–18]. Nevertheless, SARS-CoV-2 and SARS-CoV share a common ancestor with the bat HKU9-1 coronavirus [19].

Extensive sequence analysis and comparison among different animal species showed snakes to be a possible reservoir of SARS-CoV-2, and homologous recombination within the S glycoprotein possibly contributed to the snake-human transmission of SARS-CoV-2 [20], but this result remains controversial [18, 21–27]. High nucleotide identity of S protein of SARS-CoV-2—a protein with the highest genetic variations in different coronaviruses—demonstrated high homology in with two bat SARS-like coronavirus (bat-SL-CoVZX45 and bat-SL-CoVZX21). This indicates that SARS-CoV-2 might have originated from the bat, *Rhinolophus sinicus* [21, 22, 24]. Several studies have reported similar results [18, 23, 25]. More than 85% consistency of nucleotide sequence of SARS-CoV-2 has been reported with that of SARS-like coronavirus in bats (bat-SL-CoVZC45) [5, 24, 26]. The nucleotide sequence of S protein is also highly similar to that of bat-SL-CoVZC45 [5, 24, 26, 27]. The results of a study also showed that RaTG13, a short RdRp region from a bat coronavirus, is the closest relative of SARS-CoV-2, which came into light through phylogenetic analysis, and the receptor binding protein S gene was 93.1% nt identical to that of RaTG13, providing evidence to that of a bat origin of SARS-CoV-2 [21]. A recent study also revealed that pangolins might be a potential animal host of the virus [28]. The consistency of genomic sequences between beta coronaviruses isolated from pangolins and that of the currently infected human virus strains is as high as 99% [15, 29]. Metagenomic sequencing of novel pangolin coronavirus genomes demonstrated approximately 85.5% to 92.4% consistency with that of SARS-CoV-2, identifying pangolins (*Manis javanica*) as a possible host [29]. However, the natural or intermediate host is still under debate and needs further determination.

## 3. Transmission Routes

The initially infected patients had a historical exposure with the Wuhan wild animal market, and so the animal-to-human transmission should be considered during the initial phase of the pandemic [26]. According to the recent pandemic data, human-to-human transmission has evolved as a route of transmission for the virus. One family member after several days of contact with infected members was identified to be infected [26]. One person with positive throat swab in Vietnam has been reported to have close contact with his infected father [30]. A familial cluster demonstrated a close contact history with an asymptomatic carrier and developed COVID-19 [31]. An infected case was also observed outside Asia, wherein the person met a Chinese business partner with no symptoms of COVID-19; it has been suggested that the transmission might have occurred during the virus' incubation period [32]. In addition, the positive real-time reverse transcriptase polymerase chain reaction (RT-PCR) results of patients who were discharged were tested again and were shown to be viral carriers [33]. Asymptomatic carriers or positive patients should be further focused on, and their transmissibility should be reevaluated.

The nasopharyngeal and oropharyngeal swabs of COVID-19 patients were tested to be positive [4, 26, 30, 32, 34], and one positive sputum sample was also found [35] for COVID-19 by RT-PCR assay. This implies that SARS-CoV-2 particles could spread through airway. Person-to-person transmission occurs via droplets from coughing or sneezing, close contacts, or probable aerosols. Stools obtained on day 7 of illness from an infected patient in the United States also showed positive results by RT-PCR [34]. Positive RT-PCR results were also confirmed from the feces of infected patients by Guan et al. [36]. Hu et al. found that several cases showed negative results in the respiratory tract but still continued to show positive results in the stool specimens [37]. Another possible route of transmission is fecal to oral transmission, and so fecal management is also necessary. Additionally, the positive results were also obtained from conjunctival secretions and tears of an infected patient with conjunctivitis [38]. The isolation of patients and quarantining of contacts are considered essential, and hygiene protection should also be taken into account. After a 30 hour labor, a baby, whose mother was confirmed to have COVID-19, was also tested to be positive for SARS-CoV-2 in Wuhan Children's Hospital [39]. However, in another study, it was shown that the amniotic fluid, breast milk, and cord blood samples from six late pregnant patients with COVID-19 were shown to be negative [40]. At present, there is not enough evidence with regard to the identification of intrauterine infection or mother-to-child transmission.

## 4. Susceptible Population

Based on the investigation and analysis of 44,627 confirmed cases before February 11, 2020, in China, people of all ages are considered generally susceptible. Patients of 30-79 years old group were considered more susceptible, accounting for 86.6%. The cases in over 60 years old group were 31.2%. The ratio of men to women was 1.06 : 1, meaning men might be more susceptible [41]. Elderly patients with underlying diseases, such as diabetes, asthma, cardiovascular diseases, and so on, are also more susceptible. High-risk populations are those who have close contact to patients with COVID-19 [15]. Most of the pediatric patients had milder symptoms than adults, and good prognosis was also presented [42, 43]. Compared with men, women not only had fewer symptoms but also had longer incubation period. So, differential control should be adopted as early as possible [44].

Information regarding the transmissibility of COVID-19 has been published by several analytical models [45]. The WHO has estimated the *R0* (reproduction number) as 1.4 to 2.5, demonstrating that every infected person could in turn infect between 1.4 and 2.5 individuals. A series of *R0*, epidemic doubling time, incubation period, cumulative number, and case fatality rate were also calculated and analyzed [41, 46–64]. These epidemiological characteristics were estimated based on the infection in patients or even incomplete cases. These results warrant interpretation. Furthermore, stringent prevention and control measures to stop further spread or reduce transmission have been implemented in China.

## 5. Etiology and Pathogenesis

A previous study used the cells that expressed angiotensin-converting enzyme 2 (ACE2) to investigate the pathogenesis of COVID-19 and revealed that it might enter the host cells by an entry receptor ACE2 [65, 66]. Other structural analysis suggested that SARS-CoV-2 might have direct contact with ACE2 in humans [67]. Viruses enter the host cells based on the S protein attachment to the host ACE2, and the S protein on the surface of the viruses is initiated by a cellular protease [6, 16, 30, 68, 69]. Structural analysis also showed mutation of spike glycoprotein and nucleocapsid protein of SARS-CoV-2, wherein four out of five important interface amino acid residues of these were replaced but did not alter the structure, and S protein was found to have a significant attachment affinity to human ACE2 [19, 22, 66]. So, SARS-CoV-2 interacts with human ACE2 molecules, and SARS-CoV-2-S is induced by S protein during human-to-human transmission. ACE2 is widely expressed in lung AT2 cells, myocardial cells, esophagus epithelial cells, enterocytes of the ileum and colon, cholangiocytes, proximal tubular cells of the kidney, bladder urothelial cells, and spermatids or Sertoli cells [70–74]. These results indicate that the respiratory, cardiovascular, digestive, reproductive, and urinary systems might be vulnerable to COVID-19 [71, 72]. Furthermore, SARS-CoV-2 might directly bind to cholangiocytes with the ACE2 expression or ACE2 positive cells of the urinary system to induce cholangiocyte dysfunction and liver or kidney injury, respectively [72, 75]. A SARS-CoV-2 infected patient with acute respiratory distress syndrome (ARDS) demonstrated moderate lobular and portal activity and microvascular steatosis by liver histological examination [76], and also high cardiac troponin I (cTnI) was confirmed in patients with very severe infection [77]. However, no substantial histological changes in the heart tissues were seen [76], and a large number of pathological studies are necessary to confirm this conclusion.

## 6. Clinic Characteristics and Diagnosis

Detailed clinical characteristics and diagnosis were described by several articles, which were as follows (Table 1):

### 6.1. Article 1

Two adults (36 and 37 years old) had diarrhea and upper respiratory tract symptoms such as sore throat, nasal mucus, or congestion. One patient (36 years) had a productive cough. The youngest patient (10 years) with asymptomatic infection was later confirmed virologically. Three older patients (aged > 60 years) with comorbidities had more severe respiratory systemic symptoms, such as dry cough and fatigue, decreased platelet count, lymphocytes, and increased C-reactive protein. Although no bacteria existed, a secondary bacterial infection should be considered in one patient with comorbidity of chronic sinusitis based on a productive cough and high leukocyte count. The high viral loads of the lower respiratory were taken into consideration, and this is because the cycle threshold values of the sputum samples were 8–13 cycles earlier than those of throat swabs. Furthermore, repeated testing of respiratory tract samples is warranted in suspected cases [26].

### 6.2. Article 2

In this study, most of the infected patients had normal serum levels of procalcitonin. Three patients with secondary infections from Intensive Care Unit (ICU) had high levels of procalcitonin. There was a very short time of 2 days between hospital admission and ARDS, and so the mortality rate remained high. Most of the patients had some similar symptoms to that of Severe Acute Respiratory Syndrome-coronavirus (SARS-CoV) and Middle East Respiratory Syndrome-coronavirus (MERS-CoV) [78, 79], such as dry cough, fever, dyspnea, and bilateral ground-glass opacities on chest Computerized Tomography (CT) scans. A few individuals had symptoms of upper respiratory tract infection, such as rhinorrhoea, sore throat, or sneezing, which indicated SARS-CoV-2 invasion of the lower airway cells, and these were of main concern. Also, an association of cytokine storm with disease severity was found. In this article, 11 patients with productive cough and 12 patients with high white blood cell count have been reported, but there were only 4 patients with secondary infection. High white blood cell count requires no further elucidation [4].

### 6.3. Article 3

Of the 11 deaths, 8 had lymphopenia, 7 had bilateral pneumonia, 7 were >60 years, 3 had hypertension, and 3 had a long history of smoking. One study reported that smokers had higher ACE2 gene expression in lung tissues, which meant that the smokers might be more susceptible to SARS-CoV-2 infection [80]. Another study has reported that older patients who were admitted to the ICU had more comorbidities and symptoms of pharyngeal pain, dyspnea, dizziness, abdominal pain, and anorexia than non-ICU patients [81]. According to the authors' speculation, the risk of infection in men is higher, and further research should be conducted on this. In addition, 51 patients who had high interleukin-6 levels were shown to have decreased lymphocyte count suggesting that SARS-CoV-2 might mainly act on lymphocytes, especially the T lymphocytes giving rise to cascade immune responses [35].

### 6.4. Article 4

The onset of illnesses such as fever, fatigue, dry cough, myalgia, and dyspnea was reported as the most common symptoms in this study, but 14 patients presented initial and atypical symptoms, such as diarrhea and nausea. The most common laboratory abnormalities included prolonged prothrombin time, decreased total lymphocyte count, and elevated lactate dehydrogenase; these abnormal results might be associated with the activation of immune response and coagulation and injury of the myocardium, liver, and kidney. The dynamic laboratory results were also tracked in 33 patients, and the nonsurvivors had continuous increase in the levels of D-dimer, neutrophil count, blood urea, and creatinine and decrease in lymphocyte counts until the occurrence of death [81].

### 6.5. Article 5

Of the 1099 infected patients in this study, 79.1% developed pneumonia. Radiologic CT or X-ray scans appeared to be normal or observed fever as an initial symptom in only 7.55% or 43.8% cases, respectively, while diarrhea was uncommon. Signs of throat congestion were observed in 19; tonsil swelling in 23, enlargement of lymph nodes in 2, and rash in 2 were found [36]. Elevated hepatic enzymes might be correlated with the severity of infection [72]. Stool, gastrointestinal tract, saliva, or urine specimens positive to SARS-CoV-2 were detected. In one case with severe peptic ulcer, SARS-CoV-2 was directly detected at the bleeding site of esophageal erosion [36].

### 6.6. Article 6

In this article, the researchers performed dynamic observation of laboratory tests and clinical characterization of 61 patients with COVID-19. The neutrophil-to-lymphocyte ratio (NLR) was identified as an independent factor in predicting the severity of illness. Further research revealed that individuals aged ≥ 50 and NLR ≥ 3.13 contributed to the deterioration of their condition [82].

### 6.7. Article 7

Forty-five family clusters of infected individuals were included in this study in the form of 89 hospitalized patients. The symptoms in some patients with severe infection showed gradual improvement, and the abnormality of lung injury according to the chest CTs was gradually lessened after methylprednisolone and other treatments [83].

### 6.8. Article 8

In this study, a high mortality rate was observed in most of the ARDS patients with comorbidities, such as diabetes, cerebrovascular diseases, and chronic kidney diseases. The significant effect of relevant treatment plans, such as antiviral, glucocorticoid, or immunoglobulin treatments, assisted in the nonoccurrence of ARDS in patients [84].

In addition, Liu et al. have reported that COVID-19 positive patients had myalgia in 80% of cases and dyspnea in 75%. In contrast, fever and cough are the most common symptoms observed in negative patients. The combination of fever with bilaterally abnormal changes of the lung in CT scans contributed to 79.8% accuracy at initial diagnosis, and bilaterally ground-glass-like abnormality in CT scans along with the below eosinophils reached up to 77.0% [85].

During the incubation period, COVID-19 exposure history is considered to be extremely important to trace SARS-CoV-2 infection. During the onset of the disease, the infected person may present symptoms such as fever, dry cough, and fatigue, which might be accompanied by myalgia, nasal congestion, and sore throat [4, 26, 35, 36, 71–84]. However, the atypical symptoms, such as diarrhea and nausea, should also be taken into account [26, 36, 81]. Chest pain, dyspnea, long history of smoking, and comorbidities might be related to severity of illness [85, 86]. Although there are false negative patients, nucleic acid examination for COVID-19 has been the mainstay of laboratory diagnosis by viral gene sequencing or by RT-PCR from nasal and pharyngeal swabs, and repeated laboratory tests of stool, gastrointestinal tract, saliva, or urine specimens are considered beneficial.

Lung susceptibility to COVID-19 infection was observed, and this was in accordance with the results of ^18^F-FDG PET/CT imaging [87]. Typical CT imaging patterns of COVID-19 mainly included ground-glass opacification, consolidation, and ill-defined margins. The multifocal, unilateral imaging abnormalities in asymptomatic patients and ground-glass opacities have rapidly evolved into consolidation after the onset of symptoms [88, 89]. For 1,014 suspected COVID-19 patients, 88% showed positive to infection with CT scan, which was higher than 59% of RT-PCR assay in diagnosing the infected patients. Also, 42% (24/57) patients showed improvement through abnormal CT scans before confirming of the negative RT-PCR results [90]. Five patients with initially negative RT-PCR results presented ground-glass opacity and/or consolidation on CT scans, which was eventually diagnosed by repeated swab laboratory tests [91]. Also, positive CT findings showed association with time of illness onset [92, 93]. The sensitivity of CT scan was 98% during the initially infected days (i.e., 3 days) in 51 patients, while RT-PCR showed 71% sensitivity [94]. Finally, the virus mainly acted upon the lower respiratory tract to cause pneumonia [4]. CT scanning plays an important role in diagnosing and evaluating the situation of COVID-19, what at times is of great significance [95]. Also, early CT changes are considered to be a sensitive modality with which to screen patients with asymptomatic or false-negative RT-PCR results [96].

Leukocyte, lymphocyte, or platelet count were usually reduced [4, 26, 35, 36, 81–84], and progressive lymphocytopenia represents severity in cases [81]. Lymphopenia and thrombocytopenia are taken as reference indexes to diagnose COVID-19 [81, 97]. The lower levels of CD4^+^T and CD8^+^T and higher levels of cytokines, IL-6, IL-10, and so on should also be considered [85, 98, 99]. Eosinopenia is also considered as a potentially reliable laboratory indicator that should not be neglected during diagnosis [85, 100]. Additionally, the levels of CD4+T and CD8+T and IL-6, IL-10, and NLR are potentially useful for predicting the development of COVID-19 and the severity of illness [82, 101]. Abnormal coagulation function and elevated lactate dehydrogenase are considered important laboratory methods to diagnose the disease. Abnormal levels of procalcitonin might indicate secondary bacterial infection. The elevation of liver enzymes, creatine kinase, and urine protein may indicate organ dysfunction [102]. Serum specific IgM and IgG antibodies of COVID-19 have been recommended for diagnosing the disease [103]. Old age, comorbidities, and ARDS should also be taken into consideration as possible predictive risks of death [86, 104]. Failure of multiple organs, such as acute cardiac, renal, pulmonary, and hepatic injuries, still remains the main cause of death [26, 35, 36, 81]. Due to small sample size, the interpretation of relevant indicators showed individual differences.

## 7. Treatments

Currently, there is no specific antiviral therapy recommended except for the main supportive treatments. Fever is treated by using acetaminophen and ibuprofen; continued cough is treated with oxygen supplementation. In the previously published research, there are cases of distress in patients who required noninvasive or invasive ventilator mechanical ventilation (Table 1).

Most of the patients were given antiviral therapy (oseltamivir, lopinavir, ritonavir, remdesivir, nelfinavir) based on the previous reports of MERS-CoV, SARS, or HIV [105–114]. Although some possible effective antiviral drugs have been speculated on, for example, inhibitors of protease Mpro or an immunoglobulin Fc domain (ACE2-Fc), the effective proof has not been found with the use of antiviral treatment [106, 107, 114]. Remdesivir and chloroquine have been revealed to effectively control SARS-CoV-2 infection *in vitro* [110], and chloroquine has been recommended as a possible effective antiviral drug for COVID-19 in China [115, 116]. Fortunately, clinical trials on remdesivir has been approved and carried out.

Antibiotics (such as cephalosporins, quinolones, carbapenem, vancomycin, and cefepime) were given as a single or combined therapy to cover the common or atypical pathogens. Of course, for patients with secondary infection, antibiotics or antifungal drugs are essential according to the results of bacterial or fungal culture and drug sensitivity.

Some patients with rapid aggravation of chest CTs and emerging ARDS received systematic corticosteroids (methylprednisolone or dexamethasone) to reduce inflammatory-induced lung injury. However, several studies have showed that corticosteroids are an invalid treatment for lung protection and patients with SARS or MEAS [117–119]. So, corticosteroids are not routinely recommended [7, 95]. However, histological examination of biopsy samples of COVID-19 patients with ARDS showed evident desquamation of pneumocytes and hyaline membrane formation in the lungs. Also, authors suggested that timely and appropriate use of corticosteroids along with ventilator support should be considered to control ARDS development in patients with severe infection [85]. A multicentre, open-label, randomized controlled trial was used to investigate the effect of prolonged use of dexamethasone for ARDS patients and exhibited that dexamethasone showed effective reduction in the duration of ventilation and mortality of patients [120].

Continuous blood purification is regarded indispensable for renal failure [35]. Extracorporeal membrane oxygenation (ECMO) therapy is suitable when cardiopulmonary failure is difficult to cure [27]. In addition, receiving immunoglobulins strengthens the power of anti-infection during severe infectious conditions, and combined use of Chinese and Western medicine treatment is not completely excluded [108, 121].

Plasma and antibodies obtained from convalescent patients have been proposed for treatment [42]. Nebulization inhalation of human interferon *α*2b might also be an effective therapy [122, 123]. Vaccines are still under development, and the results are awaiting [124]. Now, the most available approach for infected patients is to identify them early and provide supportive treatments in a timely manner. For individuals, stringent protective precautions, such as good personal hygiene, fitted mask, and ventilation, are considered essential.

The urgent priorities for conducting the research of COVID-19 infection have been discussed in the meeting sponsored by the WHO in collaboration with GloPID-R (the Global Research Collaboration for infectious Disease Preparedness). However, significant approaches should be further identified, which are as follows. (1) Origin and intermediate transmission of vectors should be identified to stem the ongoing outbreak. The exposure history of Huanan Seafood Wholesale Market in Wuhan is still doubtful. The initially diagnosed patient has not been published in the article of 24 January 2020 [4]. Based on the phyloepidemiological analyses, the SARS-CoV-2 source at the Huanan market might be imported from elsewhere [125]. A recent or early expansion on 6 January 2020 or an expansion on 8 December 2019 was estimated according to the results of both DnaSP and Arlequin [125]. (2) The nearly identical virus sequences in different patients have suggested its recent introduction into humans, and so future surveillance of viral ability to mutate, replicate, and spread should be investigated. (3) Patients during incubation period might act as potential source of infection—in other words—there is a possibility of human-to-human transmission during the asymptomatic period [36]. Therefore, epidemiological investigation of patients is necessary and especially the history of exposure. The isolated patients and quarantining of contacts remain crucial. (4) Other transmission routes should be further determined, such as fecal-oral or vertical transmission. Also, personal hygiene and ventilation are crucial. (5) Autopsy findings of large samples are warranted to clarify the damage of the target organs and pathogenesis. (6) At present, specific drug treatment through stringent clinical trials is urgently needed to support its effectiveness and safety measures against SARS-CoV-2 infected cases.

## 8. Conclusions

The published articles were reviewed and found bat or pangolin as a probable host. Person-to-person transmission via droplets is regarded as the main mode, and the probable modes of transmission include aerosols or contacts. Older age, comorbidities, and history of smoking might assist in predicting the disease severity. Lymphopenia might be considered as a reference index to diagnose COVID-19. Lymphopenia, thrombocytopenia, abnormal coagulation function, and elevated lactate dehydrogenase levels might be taken into consideration as methods of laboratory diagnosis of COVID-19. A series of cytokines and NLR demonstrates a possibility to evaluate the severity of the illness. CT scan can be possibly used as an imaging method to diagnose and evaluate COVID-19 patients with initially negative RT-PCR or asymptomatic results. Early diagnosis and timely treatment are critical for COVID-19. It is necessary to protect important organs in patients infected with pneumonia and effective measures, such as corticosteroid treatment, which might be used to prevent the development of ARDS in severe patients. Asymptomatic or infection positive patients should also be taken into consideration. Stringent isolation of patients and tracing and quarantining of people they have been in contact with are essential to prevent further spread of COVID-19. Personal hygiene, mask precaution, and ventilation should be considered.

## Figures and Tables

**Table 1:** Clinical characteristics, laboratory tests, and treatment of included eight articles.

	Article 1 [26]	Article 2 [4]	Article 3 [35]	Article 4 [81]	Article 5 [36]	Article 6 [82]	Article 7 [83]	Article 8 [84]
Patients	6	41	99	138	1099	61	89	109
Age, y	(10-66) (range)	49 (41–58)	55.5 (13.1)	52 (42-68)	47, median	40 (1-86)	55 (23–86)	55 (43-66)
Sex (M to F)	3 : 3	30 : 11	67 : 32	75 : 63	640 : 459	31 : 30	45 : 43	59 : 50
Regions	Shenzhen City	Wuhan City	Wuhan City	Wuhan City	31 provinces/provincial municipalities	Beijing City	Shishou City	Wuhan City
Data	Dec 29, 2019-Jan 4, 2020	Jan 2, 2020	Jan 25, 2020	Jan 1-Jan 28, 2020	Jan 29, 2020	Jan 1-Jan 31, 2020	Jan 23-Feb 11, 2020	Jan 2-Feb 1, 2020
Smoking history	NA	3	NA	NA	21 ex-smokers, 137 current smokers	4 current smoking	21 current smoking	NA
Exposure history	4 Wuhan travel	27 Huanan seafood market	Huanan seafood market exposure: 47 long-term, 2 short-term	12 Huanan seafood market	483 local residents of Wuhan, 13 Huanan seafood market, 193 have been to Wuhan, 442 contacted with people from Wuhan	17 contact with infected patients, 44 Wuhan citizens or visited Wuhan	75 visited Wuhan, 45 family member infected	NA
Comorbidities	2 hypertension, 1 diabetes, 1 chronic sinusitis	8 diabetes, 6 hypertension, 6 cardiovascular disease, 1 chronic obstructive pulmonary disease, 1 malignancy, 1 chronic liver disease	40 cardiovascular and cerebrovascular disease, 11 digestive system disease, 13 endocrine system disease, 1 malignant tumour, 1 nervous system disease, 1 respiratory system disease	14 diabetes, 43 hypertension, 20 cardiovascular disease, 7 cerebrovascular disease, 4 chronic obstructive pulmonary disease, 10 malignancy, 4 chronic liver disease, 4 chronic kidney disease, 2 HIV infection	27 cardiovascular and 15 cerebrovascular disease, 23 hepatitis B infection, 10 malignancy, 8 chronic renal disease, 21 immunodeficiency, 12 chronic obstructive pulmonary disease	5 diabetes, 12 hypertension, 1 cardiovascular disease, 5 chronic obstructive pulmonary disease, 2 abnormal lipid metabolism, 1 right bundle branch block, 1 gout	10 diabetes, 15 hypertension, 3 cardiovascular disease, 5 chronic obstructive pulmonary disease, 2 malignancy, 1 chronic kidney disease, 5 chronic liver disease	12 diabetes, 37 hypertension, 7 cardiovascular disease, 4 chronic obstructive pulmonary disease, 6 cerebrovascular disease, 10 chronic kidney disease
Symptoms and signs								
Fever	4	40	82	136	473	60	86	90
Cough	4 (3 dry, 1 productive)	31	81	82	74	39	83	67
Fatigue	1	18	NA	96	419	35	30 (myalgia or fatigue)	62
Nasal congestion	1	NA	NA	NA	53	NA	NA	NA
Rhinorrhoea	1	NA	4	NA	NA	NA	NA	NA
Sneezing	1	NA	NA	NA	NA	NA	NA	NA
Sore throat	1	NA	5	24	153	10	NA	NA
Stethalgia	1	NA	2	NA	NA	1	NA	7
Headache	NA	3	8	9	150	21	15	NA
Conjunctival congestion	NA	NA	NA	NA	9	NA	NA	NA
Nausea/vomiting	NA	NA	1	14/5	55	5	NA	NA
Dizziness	NA	NA	9	13	NA	NA	16	NA
Myalgia or arthralgia	NA	18	11	48	163	NA	NA	NA
Dyspnea or shortness of breath	NA	22	31	43	204	3/7	27	NA
Anorexia	NA	NA	NA	55	NA	8	NA	NA
Sputum production	1	11	NA	37	367	27	NA	NA
Haemoptysis	NA	2	NA	NA	10	NA	NA	NA
Chill	NA	NA	NA	NA	125	12	NA	NA
Abdominal pain	NA	NA	NA	3	NA	NA	NA	NA
Diarrhea	2	1	2	14	41	6	3	12
Blood routine								
Leucocytes (×10^9^/L)	1 (↑)	6.2 (4.1–10.5), 10 (↓), 12 (↑)	7.5 (3.6), 9 (↓), 24 (↑)	4.5 (3.3-6.2)	330 (↓), 58 (↑)	4.3 (3.5-5.1)	5.9	5.2 (4.0-7.0)
Neutrophils (×10^9^/L)	1 (↑)	5.0 (3.3–8.9)	5.0 (3.3–8.1), 38 (↑)	3.0 (2.0-4.9)	NA	2.5 (2.1-3.5)	3.5	3.6 (2.8-5.6)
Lymphocytes (×10^9^/L)	2 (↓)	0.8 (0.6–1.1), 26 (↓)	0.9 (0.5), 35 (↓)	0.8 (0.6-1.1)	73 (↓)	1.0 (0.8-1.4)	0.8	0.9 (0.5-1.2)
Haemoglobin (g/L)	NA	126.0 (118.0–140.0)	129.8 (14.8), 50 (↓)	NA	134.0 (119.0–148.0)	138.0 (127.0-150.5)	NA	NA
Platelets (×10^9^/L)	2 (↓)	164.5 (131.5–263.0), 1 (↓)	213.5 (79.1), 12 (↓), 4 (↑)	163 (123-191)	315 (↓)	164.0 (135.0-219.5)	159	NA
Coagulation function								
Activated partial thromboplastin time (s)	2 (↑)	27.0 (24.2–34.1)	27.3 (10.2), 16 (↓), 6 (↑)	31.4 (29.4-33.5)	NA	NA	NA	NA
Prothrombin time (s)	NA	11.1 (10.1–12.4)	11.3 (1.9), 30 (↓), 5 (↑)	13.0 (12.3-13.7)	NA	12.0 (11.1-13.1)	NA	NA
D-dimer (mg/L)	2 (↑)	0.5 (0.3–1.3)	0.9 (0.5–2.8) (*μ*g/L), 36 (↑)	203 (121-403)	260 (↑)	NA	73	(300-1178)
Fibrinogen (g/L)	3 (↑)	NA	NA	NA	NA	NA	NA	3.1 (2.7-3.6)
Blood biochemistry								
Albumin (g/L)	NA	31.4 (28.9–36.0)	31.6 (4.0), 97 (↓)	NA	NA	44.0 (40.5-47.0)	NA	NA
Aspartate aminotransferase (U/L)	NA	34.0 (26.0–48.0),15 (↑)	34.0 (26.0–48.0), 35 (↑)	31 (24-51)	NA	NA	31	30 (21-40)
Alanine aminotransferase (U/L)	NA	32.0 (21.0–50.0)	39.0 (22.0–53.0), 28 (↑)	24 (16-40)	158 (↑)	19.0 (14.0-33.5)	34	23 (15-36)
Total bilirubin (mmol/L)	NA	11.7 (9.5–13.9)	15.1 (7.3), 18 (↑)	9.8 (8.4-14.1)	76 (↑)	NA	13	9.0 (6.5-13.4)
Blood urea nitrogen (mmol/L)	NA	NA	5.9 (2.6), 17 (↓), 6 (↑)	4.4 (3.4-5.8)	NA	4.3 (3.5-5.6)	4.9	4.3 (3.3-6.5)
Glucose (mmol/L)	NA	NA	7.4 (3.4), 1 (↓), 51 (↑)	NA	NA	6.1 (5.5-6.9)	NA	NA
Sodium (mmol/L)	1 (↓)	139.0 (137.0–140.0)	NA	NA	138.2 (136.1–140.3)	139.0 (137.0-140.0)	NA	NA
Lactate dehydrogenase (U/L)	3 (↑)	286.0 (242.0–408.0), 29 (↑)	336.0 (260.0–447.0), 75 (↑)	261 (182-403)	277 (↑)	NA	212	238 (185-341)
Creatinine (*μ*mol/L)	2 (↑)	74.2 (57.5–85.7), 4 (↑)	75.6 (25.0), 21 (↓), 3 (↑)	72 (60-87)	12 (↑)	60.0 (47.0-69.5)	76	65 (55-82)
Hypersensitive troponin I (pg/mL)	NA	3.4 (1.1–9.1), 5 (↑)	NA	6.4 (2.8-18.5)	NA	NA	NA	NA
Myoglobin (ng/mL)	NA	NA	49.5 (32.2–99.8), 15 (↑)	NA	NA	NA	NA	NA
Creatine kinase (U/L)	NA	13 (↑)	85.0 (51.0–184.0), 23 (↓), 13 (↑)	92 (56-130)	90 (↑)	93.0 (57.0-137.0)	56	91 (52-178)
C-reactive protein (mg/L)	3 (↑)	NA	51.4 (41.8), 63 (↑)	NA	481 (↑)	12.0 (3.7-27.8)	12	3.1 (1.1-5.4) (mg/dL)
Procalcitonin (ng/mL)	NA	0.1 (0.1–0.1), 3 (↑)	0.5 (1.1), 84 (↑)	49 (↑)	35 (↑)	NA	0.22	0.1 (0.1-0.2)
CT findings	6 multifocal patchy ground-glass opacities	1 unilateral pneumonia, 40 bilateral pneumonia	25 unilateral pneumonia, 74 bilateral pneumonia, 14 multiple mottling and ground-glass opacity	138 bilateral distribution of patchy shadows or ground-glass opacity	550 ground-glass opacity, 409 local patchy shadowing, 505 bilateral patchy shadowing, 143 interstitial abnormalities	48 multiple lung lobe or bilateral involvement	NA	100 bilateral distribution
RT-PCR	4 nasopharyngeal swab, 2 throat swab, 2 sputum, 1 serum	Nasal and pharyngeal swabs, bronchoalveolar lavage fluid, sputum, bronchial aspirates	Throat swab	Throat swab	Nasal and pharyngeal swab	NA	Throat swab	Throat swab
Complications								
Pneumonia	6	41	99	138	869	NA	89	NA
Acute respiratory distress syndrome	NA	12	17	NA	37	NA	NA	53
Acute cardiac injury	NA	5	NA	10	NA	NA	NA	NA
Acute kidney injury	NA	3	NA	5	NA	NA	NA	NA
Acute renal injury	NA	NA	3	NA	6	NA	NA	NA
Rhabdomyolysis	NA	NA	NA	NA	1	NA	NA	NA
Disseminated intravascular coagulation	NA	NA	NA	NA	1	NA	NA	NA
Shock	NA	3	4	12	11	NA	NA	NA
Ventilator-associated pneumonia	NA	NA	1	NA	NA	NA	NA	NA
Bacteria	NA	NA	1	NA	NA	8	NA	NA
Fungus	NA	NA	4	NA	NA	NA	NA	NA
Deaths	NA	6	11	6	15	NA	NA	31
Onset of symptom to, median (IQR), day								
Hospital admission	NA	7 (4.0–8.0)	NA	7.0 (4.0-8.0)	NA	5 (0-23), median (range)	NA	7 (5-9)
Dyspnea	NA	8.0 (5.0–13.0)	NA	5.0 (1.0-10.0)	NA	3 (2-11), median (range)	NA	NA
ARDS	NA	9 (8.0–14.0)	NA	8.0 (6.0-12.0)	37	NA	NA	NA
Median incubation period, day	NA	NA	NA	NA	3.0 (0-24), median (range)	NA	NA	NA
Treatment	NA	41 antibiotic, 38 oseltamivir, 9 corticosteroid	75 oxygen therapy, 4 invasive mechanical ventilation, 13 noninvasive mechanical ventilation, 9 CRRT, 3 ECMO, 70 antibiotic (cephalosporin, quinolone, carbapenem, tigecycline, linezolid), 15 antifungal therapy, 75 antiviral therapy (oseltamivir, ganciclovir, lopinavir, and ritonavir), 19 glucocorticoid (methylprednisolone and dexamethasone), 28 immunoglobulin	106 oxygen therapy, 17 invasive mechanical ventilation, 15 noninvasive mechanical ventilation, 4 ECMO, 13 vasopressors, 124 oseltamivir, 89 moxifloxacin, 34 ceftriaxone, 25 azithromycin, 62 glucocorticoid, 2 CRRT	418 oxygen therapy, 24 invasive mechanical ventilation, 56 noninvasive mechanical ventilation, 5 ECMO, 204 corticosteroid, 30 antifungal, 393 oseltamivir, 632 antibiotic	20 oxygen therapy, 2 invasive mechanical ventilation, 3 noninvasive mechanical ventilation, 2 corticosteroid (1 methylprednisolone, 1 methylprednisolone), 30 antifungal therapy, 34 antiviral therapy(8 oseltamivir, 26 lopinavir and ritonavir,), 26 antibiotic, 52 nebulization inhalation (human interferon *α*2b and acetylcysteine)	4 invasive and 31 noninvasive mechanical ventilation, 85 moxifloxacin, 89 interferon, 35 immunoglobulin, 35 methylprednisolone, 84 lopinaviritonavir, 4 other antibiotics, 5 other antiviral drugs	28 high-flow nasal ventilation, 106 antibiotic, 105 antiviral therapy, 72 glucocorticoid, 61 immunoglobulin

Data are median (IQR), mean (SD), and median (IQR). RT-PCR: real-time reverse transcription polymerase chain reaction; ARDS: acute respiratory distress syndrome; ECMO: extracorporeal membrane oxygenation; CRRT: continuous renal replacement therapy; NA: not applicable.

## Data Availability

N/A.
